# Hospital Sunday Fund

**Published:** 1906-12-29

**Authors:** 


### HOSPITAL SUNDAY FUND.

### ANNUAL MEETING OF CONSTITUENTS.

The Lord Mayor (Sir William Treloar) presided at the Annual General Meeting of Constituents of the Hospital Sunday Fund at the Mansion House on Thursday, December 20, and, in moving the adoption of the report of the Council, referred in sympathetic terms to the deaths of Sir Sidney Waterlow and Mr. George Herring. The latter had been good enough to bequeath a very large amount to the fund, so that they would not lose the sum he had so generously contributed for the past eight years.

The Bishop of London seconded the resolution. He always rejoiced, he said, in the meetings of that Fund, not only because of the greatness of its aims, but because also it drew together all denominations in the common bond of good work for the people of London. He had there on his right hand his friend the Chief Rabbi, whom he was always pleased to greet, and around him were the heads of the other religious communities of the metropolis. (Cheers.) If such work as that of the Fund did not draw out what was best in them, what would? He felt, with the Lord Mayor, well known for his love for children and for cripples —(cheers)—that special efforts ought to be made. He would regret it for the moral tone of London if ever the hospitals were allowed to come on the rates. But though they regretted the loss of such good friends to hospitals as Sir Sydney Waterlow and George Herring, yet, with the King's Fund raising £111,000 and their own £63,000, he thought that day was far off. They must, however, aim to get their contribution up to £100,000. (Applause.)

The Chief Rabbi supported the resolution. He said that any movement supported by the Lord Mayor and the Bishop of London needed no words from him. He also welcomed an opportunity of drawing together the various religious communities, especially at a time when certain disruptive influences were making themselves felt. He noted with pleasure the magnificent sum of £1,357 which Canon Fleming's congregation had contributed, and he was glad to see also that £322, the largest amount collected in the synagogues, was sent by the Rev. Dr. Marks, although he was now hard on his ninety-seventh year. (Applause.) He regretted that the collections in places of worship had fallen off; that undoubtedly was attributable to "week-ends," and they must exert their best efforts so that though people stayed away from places of worship they yet should not forget the sick poor. The London hospitals had patients from every county and from every colony under the British flag, and he therefore looked to the inhabitants of Greater London and of the home counties to help them to bring their contribution up to the figure at which they aimed—£100,000. (Applause.)

Mr. Henry Morris asked permission to point out what he thought were two blots on the working of that noble Fund. One was that tickets given out were stamped with a date of the year of issue, and were not accepted, even though only a few days out of date, by some of the hospitals. The other matter was that private subscribers got twice as many tickets for the amount of their subscription as did the Fund.

The Lord Mayor said the Fund had nothing to do with the conduct of individual hospitals—their duty was to raise money; he had no doubt Mr. Sydney Holland, as the London Hospital was referred to, would go into these details and explain the system.

Mr. Holland said he would be very happy to see the gentleman in the vestry after the meeting. (Laughter.)

The Bishop of Islington moved that June 9 be the date fixed for the 1907 Hospital Sunday, it being understood that June 2 would be the date so far as their Roman Catholic friends were concerned.

The Rev. Hardy Harwood, in seconding, said he ventured to make the suggestion that ministers should endeavour to secure beforehand the help of those likely to be absent on Hospital Sunday. He had learnt from Canon Fleming not to let a single individual in his congregation go unsolicited. He would also point out that they were anxious to have the help of even the smallest collections, which often represented as great a sacrifice as larger offerings. He knew from personal experience that that Fund had the confidence of the business people of London, who were glad, in charitable matters, to follow the lead of the Distribution Committee.

Canon Horsley moved that the Council for the year 1906 be re-elected for the year 1907, with the addition of the Rev. E. S. Hilliard, the Right Hon. Lord Cheylesmore, Sir Walter Vaughan Morgan, Bart., and A. Pearce Gould, Esq., F.R.C.S., M.S.; and that it be left to the Council to fill the other vacancies—one clerical, five lay members.

Canon Codrington seconded the resolution, which was supported by the Rev. Dr. Rigg. He said he had not thought he should be asked to say anything that day; he was *miles emeritus*—a worn-out soldier. But though half-way between eighty and ninety, it was a great joy and pleasure to him still to attend those meetings. He had been conected with that special movement from its very origination, when his old friend, Canon Miller, brought the idea from Birmingham to Greenwich.

Sir Savile Crossley moved a vote of thanks to the Lord Mayor, which was carried by acclamation, and the meeting separated.

